# Valedictory Address on Behalf of the Graduating Class at the Twenty-Second Annual Commencement of the Ohio Dental College

**Published:** 1868-03

**Authors:** R. J. Omahony


### DENTAL REGISTER.

VOL. XXII.] MARCH, 1868. [No. 3.

### Original Communications.

### VALEDICTORY ADDRESS ON BEHALF OF THE GRADUATING CLASS AT THE TWENTY-SECOND ANNUAL COMMENCEMENT OF THE OHIO DENTAL COLLEGE.

BY DR. R. J. OMAHONY.

MR. PRESIDENT, AND GENTLEMEN OF THE FACULTY: In the beautiful, interesting, and instructive address to which we have listened with so much attention, we are reminded that our academic course at this institution has drawn to a close, and teachers and pupils must part.

There is always more or less of sadness associated with the idea of friends parting; and the stronger the bonds that unite them, the deeper the sadness. It is not strange then that this feeling should mingle with our cheerfulness this evening, more especially as the moment approaches when the ties that have held us in pleasurable communion for the past four or five months are to be severed. Nor is it any wonder that we should feel some regret at leaving these halls, consecrated as they are to science, where for so long a time, we have sat day after day, and listened to your enlightened teachings; and I hope I may add with truth, profited by them.

But of this you must be the judges. In the Holy Gospel we learn that he who carries Christ in his heart will be known by his works. By our works ye shall know us. By our course after we have left this institution shall you know for whom, if for any, your labors have been fruitless. It may be that there are some among us for whom you have labored in vain; but when I look upon the earnest faces around me, I banish the unworthy thought, and I feel more than ever confident in my belief, that there is not one among us who has so little love for knowledge, and who has so little at heart his own honor and that of his dear old *alma mater*, as to ever allow himself to forget the principles which she has inculcated.

We are about to enter upon the practice of our adopted profession. But now we have received at your hands our diplomas which admit us to its membership. Need I say that we have received them with unfeigned pride and thankfulness? We have received them with what we hope is an excusable pride, because we believe in you and your justice; and we have received them with thankfulness, because they are, if I may so express myself, our cards of admission, and our title to membership in that honorable body of which we are proud to think you are among the staunch supports, the leading spirits, the bright and shining luminaries.

But this is not all for which we thank you. But a few years ago, comparatively speaking, Dentistry was not a science. The public mind was not prepared to accept it as such. And the public ignorance was fostered by the teachings of the medical schools, who knew of but one course of treatment, and of but one remedy for suffering Dental organisms, namely, the extraction of such *foreign* substances from the mouth where God had placed them both for use and ornament. And how this was to be effected, they knew but little and cared less. No wonder was it that scientific men saw no art in such Dental surgery, that they looked with contempt upon it, that the people detested it, and that all were content that the whole business should be monopolized by the barbers, who to-day monopolize the largest share of it in most of the countries of Europe. This is an humble origin for Dentistry; but this is nothing new. The class to whom I have alluded, seem to have a vested right in founding specialties of surgery. Prof. Brainard told the members of the American Dental Association at Chicago, that at one time operations in general surgery were performed almost exclusively by barbers. This class originated many of the instruments and appliances now in use; and as they became more and more expert in their manipulations, and more and more necessary to the community, they became more ambitious, and gained for themselves the jealousy and consequent hostility of the physicians. But the importance of surgery could not be ignored. Men of undoubted ability turned their attention to its study. The medical schools abandoned their hostility to it. And what is the position of the science to-day? Second to none—and the skilled surgeon looked upon as a benefactor of his kind. So it was; so it will be with Dentistry. In the absence of any action on the part of the medical schools, the people took up the question for themselves. They asked why those pearly beauties of the mouth should be beyond the reach of medical treatment more than any other part of the body? And they answered the query as sensible people should, and the *scienec* of Dentistry was born.

Under many difficulties and much tribulation it grew. Nursed for a long time in the lap of empiricism, it was at length adopted by scientific men, and the result is what we see to-day—Dentistry appreciated and respected from one end of the country to the other,

> "From the centre all round to the sea;—

Its professors recognized by the medical schools as practitioners in an important, nay, indispensable branch of the healing art, and numbering among them men, not mere followers, but leaders, discoverers, inventors, contributors to general science, men eminent in every branch of medical study, including that new and beautiful one which only recently was practically proved to be such an invaluable ally to the Dental Student, I mean the truly modern study of microscopy. The progress which Dentistry has made, since its inception as a science up to the present time, has no parallel in the history of the world. And to your labors, gentlemen, must this grand result be in a great measure attributed.

Our specialty, however, is by no means perfect. Though much has been accomplished, much yet remains to be done. Many fields of Dental science may yet be explored with advantage, and empiricism is still rampant.

'Tis true; 'tis pity; pity 'tis, 'tis true."

We are not unmindful of the duties and obligations which devolve upon us in our new position, the rapid progressive strides made in our profession within the last few years, only prove its youth and vigor. We know that we and the graduates of every Dental school in the country are destined to be absorbed by it, to become part of its brain and of its muscle, and as such, we must perform our functional part of thinking and acting. You have taught us how to do both; you have shown us what are the rewards of industry and patient investigation, and incited us to follow your example and inscribe upon the banners which we bear in our onward and upward course, that exalted principle, that ambitious, soul-inspiring motto, "EXCELSIOR."

These things recur to our minds at parting, for we cannot forget them; and it is for these that every student here present to-night, who has listened to your instructions during the past winter, offers you his sincere and heartfelt thanks.

Fellow Students, In addressing you on this occasion, I am moved, as doubtless you also are, by many and varied emotions. I will not enumerate them, I will not unbosom them. They are the sweet wild flowers whose pretty, fragrant blossoms greet the traveler here and there in his journey through this vale of tears, and wean him for a time from a consideration of his cares: pluck .them, and the charm is broken.

But there are some things to which allusion at this time may not be inappropriate.

As classmates, associations mutually agreeable have sprung up among us, and friendships have been formed that may last a lifetime. Associates and friends must part. Were this separation final, or were we to leave out of consideration that it is duty bids us hence, the reflection would be far from agreeable. But we have the comforting knowledge that nearly every State offers in her Dental societies many and great facilities for our meeting often and renewing the intercourse so auspiciously commenced in collegiate life. Duty and kindly feelings point us to the same course, viz.: to avail ourselves of the advantages offered to us. We owe it to ourselves and to the profession to which we belong, that we take active part in the proceedings of its societies. Our science is progressive, and we must not lag behind This is but the commencement of our labors. To stand still now would be to go backward. But our desire is to go forward and keep pace with the times, and how can we do this except by study, reading our professional and other correlative scientific literature, and attending the meetings of our professional brethren. Let no Dentist excuse himself for keeping aloof from his State society, by saying that he has nothing new to tell its members. If he has nothing to teach others, there is much that he himself may learn. All cannot be teachers, some must learn, and much is oftentimes learned from men who never pretend to foremost rank in anything. Goldsmith devotes the whole of a serious essay to a discussion of the propriety of the English nation sending an embassy of observation into Asia, in order that the most useful arts known to the people of that continent might be introduced into England. At first sight this may appear ridiculous, but a closer view will show it to be only a novel way of expressing an old idea, namely, that if we try we can learn something from everybody. You then, who say that you have nothing to tell professional brethren, reflect a little. If you only keep an account of your daily practice, it may be both useful and interesting. Boyle, the English chemist, used to say that if artists only kept a record of the observations that occurred to them while at their daily work, many and important additions would be made to philosophical knowledge.

What was true of art in general in Boyle's day is at this time especially true of Dentistry, the successful practice of which, more than that of any other science, depends upon a knowledge of little things, and it is our bounden duty to contribute as many of these *little things* as we can, to the encyclopediæ of Dental science. We must not be too conservative either in our practice as regards new inventions, and new modes; nor condemn anything until we have put it to the test. But on the other hand we must not be too precipitate in discarding old methods of practice and adopting new ones. This, I fear, is an error that is too common, and is as pernicious as it is absurd. I shall not, I hope, be suspected of a bigoted attachment to the doctrines and practices of bygone days. My creed is, that our science is in the truest sense of the word experimental, and as such it is and must be progressive. Like all other sciences it has been gradually working itself clearer and clearer, and depositing impurity after impurity. There was a time when the astrologer and the alchemist held sway over the greatest human intellects; and there was a time when the Dental *materia medica* might be comprised in one word—extraction. But time advances, facts accumulate, and doubts arise. Faint glimpses of truth appear and shine more and more into the perfect day; and out of detached facts and fragments of experience the harmonious system is at length developed. Thus the great progress goes on, and thus it will continue to go on after we are no more. But the considerations which lead us to look forward with sanguine hope to the future, should prevent us from looking back with contempt upon the past. We do not flatter ourselves with the notion that we have attained perfection, and that there is no more truth to be found. We believe that we are wiser than our ancestors. But we believe also that our posterity will be wiser than we. It would be gross injustice in those who come after us to speak of us with contempt, merely because they have surpassed us; to call Atkinson a fool, because a material for filling may be discovered which may render the use of the *mallet* unnecessary. We stand to-day upon ground which the pioneers of Dentistry could not occupy, and which it would be a scandal and a disgrace for us not to occupy. And if we bear this in mind, and bring to the examination of the results of their labors, that respectful consideration which is their due, we may discover in them much to admire, and it may be, something to imitate. Extremes are dangerous and are apt to lead to empiricism. Safety lies in the golden mean. Prove all things, hold fast that which is good.

My Brothers, the occasion which calls us together this evening is pregnant with meaning, and is as impressive as it is suggestive. Behold, here are represented the Fathers of our science, here the Grand Army of its Republic, here the documents that attest the fealty we have sworn, here the sacred volume over which we have plighted our faith.

We are now enlisted in a noble cause. Our enemy is ignorance and we must prove ourselves good soldiers if we would have honor and fortune. Like the soldier of Shakespeare's seven ages we seek reputation, and we must fight for it. But our warfare will be of a different kind. Ignorance can only be uprooted by educating the people. We must not descend to their standard, but must raise them to ours; and thus quackery, that ever panders to public ignorance, will be overpowered and crushed out of existence.

This, I am full well aware, is a delicate subject; but it is one which cannot be ignored. It will force itself upon our consideration in our every day practice, and it more or less touches the interest of each one of us. We must battle with empiricism; not, however, on the ground which *it* has chosen for the fight, but on another and a higher one of our own choosing. There are many and great temptations to go over to the enemy, but we must withstand them all and hold fast to our allegiance. Our inexperience may be sorely tried, but what of that? Let us keep in the right path, and I have faith enough in the power of truth and in the people to believe that we shall be rewarded for determined performance of duty.

Much of our success, however, will depend upon the principles which we adopt as our rule of life; but if we take honor for our guide we may be sure of safety. Let us take to heart the words of Burns to a young friend just starting out in life.

"To win Dame Fortune's golden smile
Assiduous wait upon her;
And gather gear by every wile
That's justified by honor.

Not to hide it in a hedge,
Not for a train attendant;
But for the glorious privilege
Of being independent.

The fear o' hell's a hangman's whip
To haud the wretch in order,
But where you find your honor grip
Let that aye be your border.

It's slightest touches—instant pause,
Debar aside pretences;
And resolutely keep its laws,
Uncaring consequences."

My Brothers, allusion has been made to a subject that touches the heart of each one of us, the death of one of the most industrious and most respected members of our class, Dr. Alfred A. Sears. I need not tell *you* with what profound sorrow we received the sad intelligence, nor how our hearts with one accord vibrated in one thrill of sympathy for his bereaved parents. They saw him depart from his home for college in all the pride of early manhood, only to behold their beloved again struggling in the grasp of relentless death, powerless to save him, and too agonized with grief to be comforted with the assurance that he was leaving a world of misery for one of eternal happiness. Let us draw a veil over their grief.

> "And in lonely silence shed one tear
> In mourning over this untimely bier."

Respected Professors, In the name of the graduating class I bid you farewell. You remain to continue the work in which you have labored so faithfully and so well. May God give you strength to continue your labors for the advancement of science. Unfortunately, Dentistry is something like republics as regards gratitude; but have at least the satisfaction of knowing that the present class will carry with them from this institution the most grateful recollections of the session of 1867–'8.

Fellow Students, I bid you farewell; but not for ever. Although the different fields of labor for which we set out are widely scattered, yet the roads leading from them, some long, some short, converge to meet in that abode of bliss where I trust I shall see you all again.

> "Adieu, adieu, is often said
> With a tear and perhaps with a sigh;
> But give to me that good old word
> That comes from the heart, "Good Bye.' "

